# Nutritional status in patients with neuro-Behçet’s disease

**DOI:** 10.3906/sag-2009-235

**Published:** 2021-08-30

**Authors:** Seyda ERDOĞAN, Mine Hayriye SORGUN, Müge KUZU MUMCU, Mustafa Aykut KURAL, Sefer RZAYEV, Canan YÜCESAN

**Affiliations:** 1 Department of Neurology, Faculty of Medicine, Ankara University, Ankara Turkey; 2 Department of Neurology, Faculty of Medicine, Aarhus University, Aarhus Denmark

**Keywords:** Behçet’s disease, neuro-Behçet’s disease, malnutrition

## Abstract

**Background/aim:**

The aim of this study was to assess the nutritional status and risk factors for malnutrition in Behçet’s disease (BD) and neuro-Behçet’s disease (NBD) patients.

**Materials and methods:**

Medical recordings of 74 patients with BD without neurological involvement (BDWoNI), 72 patients with NBD, and 50 patients with other diseases (carpal tunnel syndrome or lumbar discopathy) were analyzed retrospectively. The serum analyses were performed in the inactive period of disease. Chronic malnutrition was defined as low levels of serum albumin (<3.5 g/dL) with normal sedimentation rate and normal serum CRP levels.

**Results:**

Six (8.3%) of the patients in NBD group, 1 (1.4%) of the patients in BDWoNI group, and none of the patients in control group had chronic malnutrition (p = 0.029). Malnutrition rate was higher in NBD than control group (p = 0.036). The mean expanded disability status scale score was 2.92 ± 3.35 (range: 0–8) in patients with malnutrition and 1.44 ± 1.76 (range: 0–9) in patients without malnutrition in NBD group (p = 0.457). The ratio of patients having a progressive disease course was 33.3% and 7.6% in patients with and without malnutrition in NBD group, respectively. The median value of the duration of neurological involvement was 2 years (0–16) in patients with malnutrition and 6.5 years (0–18) in patients without malnutrition in NBD group (p = 0.046). There was no association between malnutrition and medications, disability scores, functional system involvement or findings on brain MRI.

**Conclusion:**

Malnutrition was higher in patients with NBD. Higher disability level and progressive disease course may be risk factors for malnutrition in NBD. Malnutrition may be seen more frequently in earlier phases of neurological involvement.

## 1. Introduction: 

Behçet’s disease (BD) is a multisystem immune mediated disorder of unknown etiology. Nervous system involvement in BD is referred to as neuro-Behçet’s disease (NBD) and classified as parenchymal and nonparenchymal [1]. Brainstem, thalamus, basal ganglia, spinal cord, and cerebral hemispheres are main regions affected in parenchymal NBD. Manifestations of nonparenchymal NBD include cerebral venous thrombosis, intracranial hypertension syndrome (pseudotumour cerebri), and aseptic meningitis [1,2].

Malnutrition can result from starvation, diseases or advanced ageing, alone or in combination [3]. Malnutrition in chronic inflammatory diseases such as systemic lupus erythematosus (SLE), rheumatoid arthritis (RA), and systemic sclerosis and in which gastrointestinal system is predominantly affected such as Crohn’s disease or ulcerative colitis has been investigated in several studies [4–8]. However, malnutrition rate and risk factors for malnutrition and impact of nutritional status on the course of disease have not been well studied in chronic inflammatory disorders affecting nervous system. To our knowledge there is no study in English based literature investigating nutritional issues in NBD, a relatively rare inflammatory disease of nervous system. The aim of this study is to assess the nutritional status and risk factors for malnutrition in NBD patients. 

## 2. Materials and methods

Approval was obtained from the research ethics committee of Ankara University. Files of the patients with a diagnosis of Behçet’s disease and neuro-Behçet’s disease between January 2007 and December 2018 at our hospital were reviewed. As a control group, patients with carpal tunnel syndrome or lumbar discopathy who had serum albumin, C-reactive protein (CRP) levels and erythrocyte sedimentation rate in their files were included in the same period. Date of diagnosis with BD and NBD, type of NBD (parenchymal or nonparenchymal), medications, functional system involvements according to the Kurtzke functional system score, expanded disability status scale (EDSS) scores, modified Rankin scale score (mRS), findings on brain magnetic resonance imaging (MRI), serum albumin, C-reactive protein (CRP) levels, and erythrocyte sedimentation rate that were performed in the inactive period of BD and NBD were recorded. Just serum albumin, C-reactive protein (CRP) levels and erythrocyte sedimentation rate were documented in the control group. Chronic malnutrition was defined as low levels of serum albumin (<3.5 g/dL) with normal sedimentation rate and normal serum CRP levels [3]. 

Statistical analysis was performed using the Statistical Package for the Social Sciences v: 11.5 (SPSS, Chicago, IL, USA). Data are presented as mean ± standard deviation and median (minimum-maximum) for quantitative variables, and as percentages for qualitative variables. Chi-Square test was used for comparing qualitative variables between groups. 

When normal distribution is not the case, Mann–Whitney U and Kruskal–Wallis tests were used for comparison of quantitative variables in two and more than two groups, respectively. When normal distribution was provided, the differences of quantitative variables between more than two groups were analyzed using a one way ANOVA test. A value of p < 0.05 was considered statistically significant. When Kruskal–Wallis was applied, Bonferroni correction was applied for multiple comparisons, and a p value < 0.016 (0.05/3) was considered significant as error correction. 

## 3. Results

Seventy-two patients with NBD, 74 patients with BD without neurological involvement (BDWoNI), and 50 patients with other diseases as control group (carpal tunnel syndrome or lumbar discopathy) were included in the study (Table 1). The mean ages of patients in the groups were similar (ANOVA, p = 0.419). As the result of the ANOVA test was not significant, multiple comparisons were not done as further analysis. The ratio of male patients was higher in the NBD group (61.1%, p = 0.01); whereas male/female ratio was similar in other two groups (p = 0.115). Duration of Behçet’s disease was longer in NBD group (p = 0.002). Data about neuro-Behçet’s disease duration, NBD type, EDSS score and mRS scores were available for only NBD patients. Demographic and clinical features of the patients are summarized in Table 1.

**Table 1:** Demographic and clinical features of patients.

		NBDn = 72	BDWoNIn = 74	Controln = 50	P
Age, years, mean ± SD		46.46 ± 11.66	43.73 ± 13.06	45.64 ± 13.69	0.419a
Sex, n(%)					
	Female	28(38.9)	43(58.1)	36(72)	0.001*b
	Male	44(61.1)	31(41.9)	14(28)	
BD duration, median (min-max)		14(0–42)	8.5(0–30)		0.002*c
NBD duration, mean ± SD		6.31 ± 4.05			
NBD typeparenchymal, n(%)CVT, n(%)Parenchymal + CVT, n(%)PTC, n(%)		81(80)47(65.3)16(22.2)5(6.9)4 (5.6)			
EDSS Mean ± SD Median (min-max)		1.56 ± 1.951(0–9)			
mRS Mean ± SD Median (min-max)		1.42 ± 1.241(0–5)			
Albumin (g/dL), Median(min-max)		4.1(2.5–5.7)	4.3(3.3–5.0)	4.1(3.5–5.1)	<0.001*d
Malnutrition, n(%)		6(8.3)	1(1.4)	0(0)	0.029*b

The disease was inactive in all patients in BDWoNI and NBD groups when blood analysis was performed. Mean serum albumin levels were 4.1(2.5–5.7), 4.3(3.3–5.0), 4.1(3.5–5.1) g/dL in NBD, BDWoNI and control group, respectively. Mean serum albumin level was higher in the BDWoNI group than the other two groups (p < 0.001). Six (8.3%) patients in the NBD group, 1 (1.4%) patient in the BDWoNI group had chronic malnutrition whereas there was not any patient with chronic malnutrition in the control group; the malnutrition rate was different statistically between three groups (p = 0.029); the difference was between NBD and control group (p = 0.036); malnutrition rate in NBD was similar to the BDWoNI group (p = 0.061). 

In the NBD group, the mean EDSS score was 2.92 ± 3.35 (range: 0–8) in patients with malnutrition and 1.44 ± 1.76 (range: 0–9) in patients without malnutrition; the difference was not statistically significant (p = 0.457). The NBD type was parenchymal in 43 (65.2%) of patients without malnutrition and 4 (66.7%) of patients with malnutrition; the difference was not statistically significant (p = 0.843). Two of six patients with malnutrition in NBD group (33.3%) had a progressive course; this ratio was 7.6% (5 of 66 patients) in the group of NBD patients without malnutrition; the difference was not significant (p = 0.101). Eight patients in NBD group and 6 patients in BDWoNI group had gastrointestinal system (GIS) involvement; interestingly, none of them had malnutrition.

The median value of BD duration was 20.5 years (min-max: 0–25) in patients with malnutrition and, 14 years (min-max: 1–42) in patients without malnutrition in NBD group; the difference was not significant (p = 0.909). Due to presence of only one patient with malnutrition in BDWoNI group, statistical analysis was not possible for evaluation the relationship between disease duration and malnutrition in that group. So, we collected all patients with (6 patients in NBD group and one patient in BDWoNI group) and without malnutrition (66 patients in NBD group and 73 patients in BDWoNI group) in two groups, and assessed whether BD duration had any effect on the presence of malnutrition; there was not any relationship between them (p = 0.804). The median value of the duration of neurological involvement was 2 years (0–16) in patients with malnutrition and 6.5 years (0–18) in patients without malnutrition in NBD group; malnutrition rate was higher in patients with shorter duration of neurological involvement (p = 0.046). EDSS score, mRS score, functional system involvement (pyramidal, brainstem, visual, mental, cerebellar, sensory, bowel and bladder functions) and lesion localization on brain MRI were not related to the presence of malnutrition in NBD group (Table 2). 

**Table 2:** Clinical/treatment features and lesion localizations on MRI of patients.

	NBD group		BDWoNI group		
	NBWMn = 6	NBWoMn = 66	BDWM n = 1	BDWoMn = 73	p
BD duration, median (min-max)	20.50(0–25)	14(1–42)	11	8.5(0–30)	0.909a
Duration of neurological involvement in NBD group median (min-max)	2(0–16)	6.5(0–18)			0.046*b
Azatiopyrin (%)Steroid (%)Colchium (%)	5 (83)2 (33)4 (67)	37 (56)12 (18)52 (79)	1 (100)0 (0)1 (100)	14 (19)2 (3)69 (94.5)	0.291c0.364c0.429c
GIS involvement (%)	0 (0)	8 (12)	0 (0)	6 (8)	0.325c
Progressive course (%)	2 (33)	5 (8)			0.101c
EDSS, median (min-max)mRS, median (min-max)	2.5 (0–8)3 (0–5)	1.25 (0–6)1(0–4)			0.460b0.250b
Lesion on MRI (%)MesencephalonPons BulbusBrainstemThalamusCerebellumFrontalParietal OccipitalTemporalHemisphere	2 (33.3)2 (33.3)0 (0.0)2 (33.3)0 (0.0)1(16.6)0 (0.0)0 (0.0)0 (0.0)0 (0.0)0(0.0)	23 (34.8)26 (39.4)11 (16.6)28 (42.4)9 (13.6)4 (6.1)12 (18.2)14 (21.2)6 (9.1)9 (13.6)17 (31.5)			0.657c0.549c0.342c0.484c0.423c0.368 c0.306 c0.244 c0.573 c0.423 c0.170 c
FSI (%)PyramidalSensoryBrainstemVisualMentalCerebellarBowel and bladder	3(50.0)1(16.6)2 (33.3)2 (33.3)0 (0.0)2 (33.3)0 (0.0)	31 (46.9)26 (39.4)16 (24.2)11 (16.6)5 (7.6)19 (28.8)6 (9.1)			0.608 c0.263c0.470 c0.295 c0.639 c0.259 c0.582 c

(a) Kruskal–Wallis; (b) Mann–Whitney U; (c) Chi-Square.

Except one patient in BDWoNI, all patients in BDWoNI and NBD group were treated with azatiopyrin and/or kolsisin (Table 2). Oral steroid plus azatiopyrin and/or kolsisin were given in three and 14 patients in BDWoNI group and NBD group, respectively; there was not any significant difference between patients with and without malnutrition in terms of treatment (p = 0.291, p = 0.364, p = 0.429 for steroid, azatiopyrin, and kolsisin respectively). 

## 4. Discussion

It has been well known that neuro-Behçet’s disease is more frequent in males [1]. A similar finding was found in our NBD group, too. Behçet’s disease may involve gastrointestinal system which may have a major effect on malnutrition. There are some data on malnutrition in inflammatory bowel diseases (IBD). In a large prospective multicenter study, the prevalence of malnutrition was 20% in outpatients with IBD [8]. In that study, the risk factors for malnutrition were active disease, history of abdominal surgery and self-imposed food restrictions. The authors emphasized the high frequency of food avoidance in patients with IBD based on their beliefs. There were 12 patients with gastrointestinal system involvement in our study, the disease was inactive at the time that serum samples were obtained, and none of these patients had chronic malnutrition. This finding may indicate that gastrointestinal involvement unless it is active, has no major contributing effect on chronic malnutrition in BD patients. 

The mechanisms in disease related malnutrition without inflammation include dysphagia, psychiatric disorders and malabsorption. Inflammation is known to have an important role in malnutrition [9–11]. The pathways triggered by inflammation are different, so the term ‘disease-related malnutrition with inflammation (DRMWI)’ is used [9]. Inflammation leads to catabolic condition and results in anorexia and tissue breakdown. Disease related malnutrition with inflammation is classified as chronic DRMWI and acute disease- or injury-related malnutrition. There is a more prominent inflammatory response in acute DRMWI. On the other hand, chronic DRMWI may be seen in patients with a chronic disorder including cancer, chronic kidney and pulmonary diseases or congestive heart disease and is characterized by malnutrition and an ongoing mild inflammatory activity which is indicated by biochemical markers including high levels of serum CRP and/or low levels of serum albumin [9]. According to this definition, reduced levels of albumin, even in the case of normal levels of serum CRP is considered as an indicator of inflammation. In other words, inflammation is regarded as a cause of malnutrition [10]. In the last ESPEN guideline, the authors mentioned that there are no good biochemical markers of nutritional status, and plasma albumin levels may be used to monitor catabolic activity [9]. The levels of serum albumin, prealbumin, transferrin, retinol binding protein, complement C3 fraction and immunoglobulin M have been widely used as indicators of nutritional status [12–14]. Among these serum proteins, albumin has been considered as a better indicator for chronic malnutrition because of its long half-life (21 days) [13,14]. Measurement of serum albumin level is easy and has a low cost. Based on some previous studies, we defined chronic malnutrition as low levels of serum albumin with normal sedimentation rate and normal serum CRP levels [13,14].

Chronic malnutrition rate was higher in patients with NBD (8.3%) than control group (0), in the study. Moreover, although the difference was not statistically significant, chronic malnutrition rate was higher in NBD group than BDWoNI group (1.4%). We thought the involvement of nervous system may be a major risk factor for malnutrition. On the other hand, Baron M et al. demonstrated that although more than 20% of their systemic sclerosis cohort was at high risk for malnutrition, only 2% of them had low albumin levels; they suggested that low serum albumin level may not be a useful marker for malnutrition because patients with malnutrition may have normal albumin levels in chronic diseases [6]. This issue should be clarified through further research. 

The nutritional profile of patients with chronic inflammatory disorders including rheumatoid arthritis (RA), systemic lupus erythematosus (SLE), and systemic sclerosis may be affected by the inflammation with altering the metabolism and by drugs used to control the disease which causes appetite and gastrointestinal changes [4,7]. Limited physical activity due to joint involvement also may contribute to malnutrition in these patients. A study assessing the nutritional status of 72 women with rheumatoid arthritis using anthropometric and food consumption measurements showed that, despite half of the patients were overweight or obese, their calorie and microelements intake were low [4]. Moreover, the frequency of low-calorie intake was higher among patients treated with combined leflunomide+methotrexate and leflunomide+prednisone. Fukuda et al. showed that the mean arm muscle area (which expresses muscle protein) was significantly lower and serum albumin level (which expresses visceral protein) was negatively correlated with disease activity in patients with RA [5]. Although we did not find any effect of BD duration on the presence of malnutrition, the patients with malnutrition had longer disease duration than without it in all BDWoNI and NBD groups. However, we found that the duration of neurological involvement was shorter in patients with malnutrition than others in NBD group. We thought that malnutrition may be more frequent in earlier years of neurological involvement and with good control of the disease the rate of malnutrition may decrease with time. Moreover, the treatment modalities were similar in patients with and without malnutrition. Therefore, we thought the medications have no any effect on malnutrition in NBD patients.

Malnutrition rate and risk factors for malnutrition have been reported in patients with stroke, multiple sclerosis (MS), amyotrophic lateral sclerosis (ALS), and Parkinson’s disease (PD). For instance, a meta-analysis revealed the risk factors for malnutrition in stroke as malnutrition on admission, dysphagia, previous stroke, diabetes mellitus, tube feeding and reduced level of consciousness [15]. Among these risk factors, dysphagia is considered to have a major contributing effect on malnutrition. Recently, the European Society for Clinical Nutrition and Metabolism (ESPEN) reported a guideline for nutrition in neurology [16]. This guideline offers recommendations for malnutrition in patients with stroke, ALS, MS and PD, and is generally focused on dysphagia. However, malnutrition may not always be caused by dysphagia. It has been shown that there was no association between dysphagia and malnutrition in MS patients [17,18]. In our study, too, none of our patients had dysphagia and there was not any association between brainstem involvement and malnutrition in NBD patients. Despite the difference was insignificant, the ratio of patients having a progressive neurological disease course was higher in patients with malnutrition than in patients without malnutrition (33.3% vs. 7.6%, respectively) in NBD group in the study. The underlying inflammatory mechanisms may differ between relapsing remitting and progressive disease course. 

It has been shown that olfactory function may be impaired in BD patients [19]. Olfactory dysfunction may affect appetite and cause malnutrition. However, we did not evaluate olfactory functions in the study; this issue should be investigated in future researches. 

Mean EDSS was almost 3 and 1.5 in patients with and without malnutrition in NBD group, respectively. Although the difference was not significant statistically, we thought higher disability level may negatively affect malnutrition frequency; this issue should be evaluated in future studies including larger sample sizes. 

Another aspect of malnutrition in NBD may be mood disorders. It has been shown that total energy intake was significantly higher in patients with stroke that not having anxiety/depression than in patients having anxiety/depression [20]. Mood disorders are common in BD patients and may alter food intake [21].

Six (8.3%) of patients in NBD group, 1 (1.4%) of patients in BDWoNI group, and none of the patient in control group had chronic malnutrition (p = 0.029). Malnutrition rate was higher in NBD than control group (p = 0.036). The mean EDSS score was 2.92 ± 3.35 (range: 0–8) in patients with malnutrition and 1.44 ± 1.76 (range: 0–9) in patients without malnutrition in NBD group (p = 0.457). The ratio of patients having a progressive neurological disease course was 33.3% and 7.6% in patients with and without malnutrition in NBD group, respectively. The median value of the duration of neurological involvement was 2 years (0–16) in patients with malnutrition and 6,5 years (0–18) in patients without malnutrition in NBD group (p = 0.046). There was no association between malnutrition and medications, disability scores, functional system involvement or findings on brain MRI.

In summary, the rate of chronic malnutrition was higher in NBD than control group in this study. Moreover, despite it has not been found statistically significant, chronic malnutrition rate was higher in NBD group. Higher disability level and progressive disease course may be risk factors for malnutrition in NBD. Furthermore, malnutrition may be seen more frequently in earlier phases of neurological involvement. 

Our study was retrospective in nature. NBD is a relatively rare neurological disorder, so, the number of patients was small. Chronic malnutrition was defined with only serum albumin and CRP levels and sedimentation rate in the study. A detailed nutritional assessment was not performed. For these reasons the results of this study should be interpreted cautiously. However, findings of this study may draw attention to the higher incidence and potential importance of nutritional status in patients with NBD. There seems to be a number of possible risk factors needed to be investigated including possible taste and odor changes, depression/anxiety presence, dietary habits, and their effect on nutritional status in patients with NBD. Future prospective studies including larger sample sizes may provide a better understanding of nutritional status and risk factors of malnutrition in NBD patients. 

## Informed consent

The study was a clinical study and retrospective in nature. So, we did not provide informed consent. 
